# Supplementary material for: Complexome profiling of the Chlamydomonas psb28 mutant reveals TEF5 as an early PSII assembly factor
Source: Plant Cell. 2025 Mar 17;37(6):koaf055. doi: 10.1093/plcell/koaf055 (PMC12164590; doi:10.1093/plcell/koaf055)
Supplement: koaf055_Supplementary_Data [file koaf055_supplementary_data.zip › psb28_interactive.zip!/plots/intensity/qualityPlots/peptides/_Cre13.g586050.t1.1.html]

### Cre13.g586050.t1.1

Trivial name:   
SeqCoverage [median]: 21.70  
Mass: 79.71 kDa  
  
 

| Sequence | MissedCleavage | Mass [Da] | wtIntensity | muIntensity | Score |
| --- | --- | --- | --- | --- | --- |
| AIDLTTK | 0 | 760.43 | 0.00 | 1648.48 | 74.42 |
| ALNPNTNEITTLAGGGAAGFR | 0 | 2044.03 | 0.00 | 1722.75 | 51.82 |
| DGSFEMAALNRPQGLAYSPR | 1 | 2179.05 | 2502.56 | 5100.61 | 102.58 |
| EAAFSEPAGLAR | 0 | 1217.60 | 4386.13 | 5967.73 | 140.05 |
| FEVVPAAQTSFVLQ | 0 | 1534.80 | 1122.99 | 0.00 | 91.55 |
| GPGGTVLVADTNNAAIR | 0 | 1624.85 | 3218.71 | 3953.69 | 122.34 |
| LFVSDSNNHR | 0 | 1187.57 | 1766.17 | 0.00 | 77.19 |
| NTLYVADTENHAVR | 0 | 1601.78 | 2873.30 | 1154.41 | 59.86 |
| VDLPAGYR | 0 | 889.47 | 1036.13 | 0.00 | 62.72 |
| VGGDPLFSDNLFR | 0 | 1435.71 | 2604.40 | 0.00 | 100.72 |
| VLDLSSGGSGLK | 0 | 1131.61 | 3231.91 | 7968.86 | 101.38 |
| VVITDLAGNFIEAVGGNGPALR | 0 | 2182.17 | 845.38 | 0.00 | 62.65 |
|  |  |  | 23587.7 | 27516.5 |  |
